# Development and Assessment of a 3D-Printed Scaffold with rhBMP-2 for an Implant Surgical Guide Stent and Bone Graft Material: A Pilot Animal Study

**DOI:** 10.3390/ma10121434

**Published:** 2017-12-16

**Authors:** Ji Cheol Bae, Jin-Ju Lee, Jin-Hyung Shim, Keun-Ho Park, Jeong-Seok Lee, Eun-Bin Bae, Jae-Won Choi, Jung-Bo Huh

**Affiliations:** 1 Department of Prosthodontics, Dental Research Institute, Institute of Translational Dental Sciences, BK21 PLUS Project, School of Dentistry, Pusan National University, Yangsan 50612, Korea; inteliron@hanmail.net (J.C.B.); ljju1112@hanmail.net (J.-J.L.); 0228dmqls@hanmail.net (E.-B.B.); won9180@hanmail.net (J.-W.C.); 2 Department of Mechanical Engineering, Korea Polytechnic University, 237 Sangidaehak-Ro, Siheung 15073, Korea; happyshim@kpu.ac.kr (J.-H.S.); rootpark153@gmail.com (K.-H.P.); suk3301@naver.com (J.-S.L.)

**Keywords:** 3D-printed scaffold, rhBMP-2, surgical guide

## Abstract

In this study, a new concept of a 3D-printed scaffold was introduced for the accurate placement of an implant and the application of a recombinant human bone morphogenetic protein-2 (rhBMP-2)-loaded bone graft. This preliminary study was conducted using two adult beagles to evaluate the 3D-printed polycaprolactone (PCL)/*β*-tricalcium phosphate (*β*-TCP)/bone decellularized extracellular matrix (bdECM) scaffold conjugated with rhBMP-2 for the simultaneous use as an implant surgical guide stent and bone graft material that promotes new bone growth. Teeth were extracted from the mandible of the beagle model and scanned by computed tomography (CT) to fabricate a customized scaffold that would fit the bone defect. After positioning the implant guide scaffold, the implant was placed and rhBMP-2 was injected into the scaffold of the experimental group. The two beagles were sacrificed after three months. The specimen block was obtained and scanned by micro-CT. Histological analysis showed that the control and experimental groups had similar new bone volume (NBV, %) but the experimental group with BMP exhibited a significantly higher bone-to-implant contact ratio (BIC, %). Within the limitations of this preliminary study, a 3D-printed scaffold conjugated with rhBMP-2 can be used simultaneously as an implant surgical guide and a bone graft in a large bone defect site. Further large-scale studies will be needed to confirm these results.

## 1. Introduction

Restoration of the edentulous arch with a dental implant is becoming a predictable treatment option, and various prosthodontic treatment approaches have been developed and introduced [1]. Early implant research focused on the osseointegration. On the other hand, most current studies have shifted focus to the functional and esthetic restoration of the implant with a minimally invasive surgical procedure. Digital technology including computer-aided design (CAD), cone-beam computed tomography (CBCT), and rapid prototyping (RP) has improved, and these technologies have been brought together to introduce digital-guided surgery technology. Digital-guided surgery has the potential to be an accurate and simplified surgical implant procedure [2]. Moreover, these techniques can be used to perform flapless surgery, which is less invasive than conventional implant surgery, and it reduces the post-operative discomfort and healing period [3,4]. Nevertheless, computer-guided surgery still has to overcome anatomical obstacles, especially for the clinical cases that require vertical and horizontal bone graft procedures [5,6]. A longer treatment period is needed for the clinical cases that require a bone graft because the subsequent procedure is delayed until successful ossification of the grafted material has been achieved.

Several studies have investigated a range of surgical procedures for bone grafts and ideal bone graft substitutes. The optimal bone graft substitute requires the following properties: (1) bioresorbable; (2) biocompatible; (3) enhanced proliferation of bone-forming cells; (4) osteoconductive and osteoinductive properties; (5) physically and chemically like bone; (6) provide calcium and phosphate; (7) microporous; (8) easy to use; and (9) space-maintaining ability and biomechanical stability during the early healing period [7]. Autogenous bone graft material is the most predictable substitute that exhibits both osteoinductive and osteoconductive properties. On the other hand, additional augmentation surgery is required at the donor site and the procedure causes post-operative pain and discomfort, as well as an additional healing period and cost [8,9]. Bone substitutes with osteoconductivity provide a favorable environment for the penetration of blood vessels and the formation of a scaffold that promotes new bone growth; however, effective bone regeneration requires at least three-walled bony defects [8,9].

In the field of bone tissue engineering, a 3D-printed polymeric scaffold has attracted recent attention due to its biomechanical properties in the repair of large bone defects that are otherwise difficult to restore using the conventional procedure [10,11]. A bone repair scaffold improves the osteoconductive properties of cells and growth factors. On the other hand, poor angiogenesis may cause localized necrosis and implant failure [12,13,14,15]. Reconstruction of various bone defects, controlling the pore size, porosity and geometry of the scaffold, and the elimination of toxic solvents are important considerations when producing a 3D scaffold [16]. The multi-head deposition system (MHDS) is one type of solid freeform fabrication (SFF) technology for effectively producing a 3D scaffold—using polycaprolactone (PCL) and *β*-tricalcium phosphate (*β*-TCP)—for various bone defects [17,18]. Furthermore, target-specific scaffolds with cells or growth factors have been actively studied. Recombinant human bone morphogenetic protein-2 (rhBMP-2) is a growth factor with powerful osteoinductive properties in bone formation and repair [19]. Although rhBMP-2 is considered as the most promising growth factor, its application is still limited for several reasons: toxic effects, release rate, encapsulation of the cell, and cytocompatibility as a printing material [20,21,22,23,24].

Materials such as PCL, one of the most commonly used biomaterials, undergo a high-temperature polymer melting process for 3D printing and the elevated temperature can easily cause denaturation of living cells. Bioink is an ink formulation that is used in tissue engineering to convert living cells into cytocompatible inks [25]. A range of bioinks including cell-laden hydrogels, decellularized extracellular matrix (dECM)-based solutions, and cell suspensions are used to load living cells into biomaterials [26,27,28]. A previous study assessed a PCL-based 3D scaffold and bone decellularized extracellular matrix (bdECM) as a bioink and reported significant improvement in bone tissue formation at the bone defect site [29]. Moreover, bdECM provided numerous types of anchoring sites, promoted osteogenesis, and increased the concentrations of bone morphogenetic protein (BMP)-2 and BMP-7 when it was used as a bioink [30].

In this study, an innovative concept of a 3D-printed scaffold was designed to overcome the limitations of digital-guided surgery. The proven osteoconductive 3D-printed PCL/*β*-TCP/bdECM scaffold was placed at the vertical bone defect and the implants were simultaneously placed using the surgical guide holes that had been designed (using software) and drilled into the scaffold. After the implant had been inserted, rhBMP-2 was injected into the scaffold intraorally. This pilot animal study, conducted on a beagle dog model, was a preliminary evaluation of the ability of a 3D-printed scaffold to promote new bone growth, as well as its effectiveness as a surgical guide and its potential in future clinical applications.

## 2. Results

### 2.1. In Vitro Results

#### 2.1.1. Morphology of a Fabricated Implant Guide Scaffold Using an In-House System

**Figure 1:**
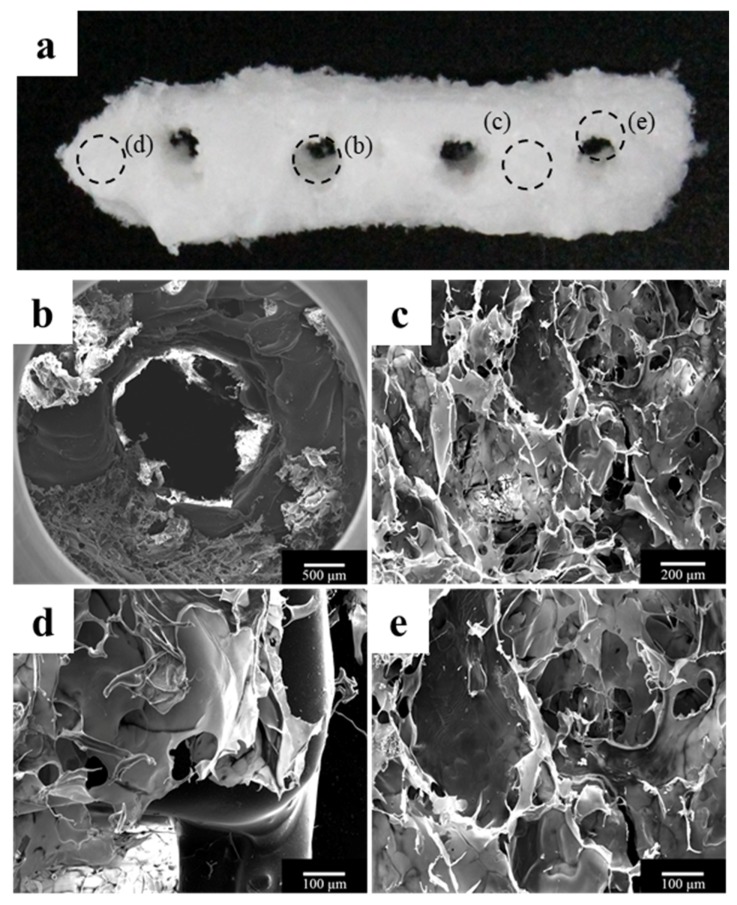
Morphology of the polycaprolactone (PCL)/β-tricalcium phosphate (β-TCP)/bone decellularized extracellular matrix (bdECM) scaffold. (**a**) Visual image of the scaffold; (**b**) An implant through hole, which plays a role in guiding the implant fixture and (**c**–**e**) Bone decellularized extracellular matrix (bdECM) coated on the scaffold.

**Figure 2:**
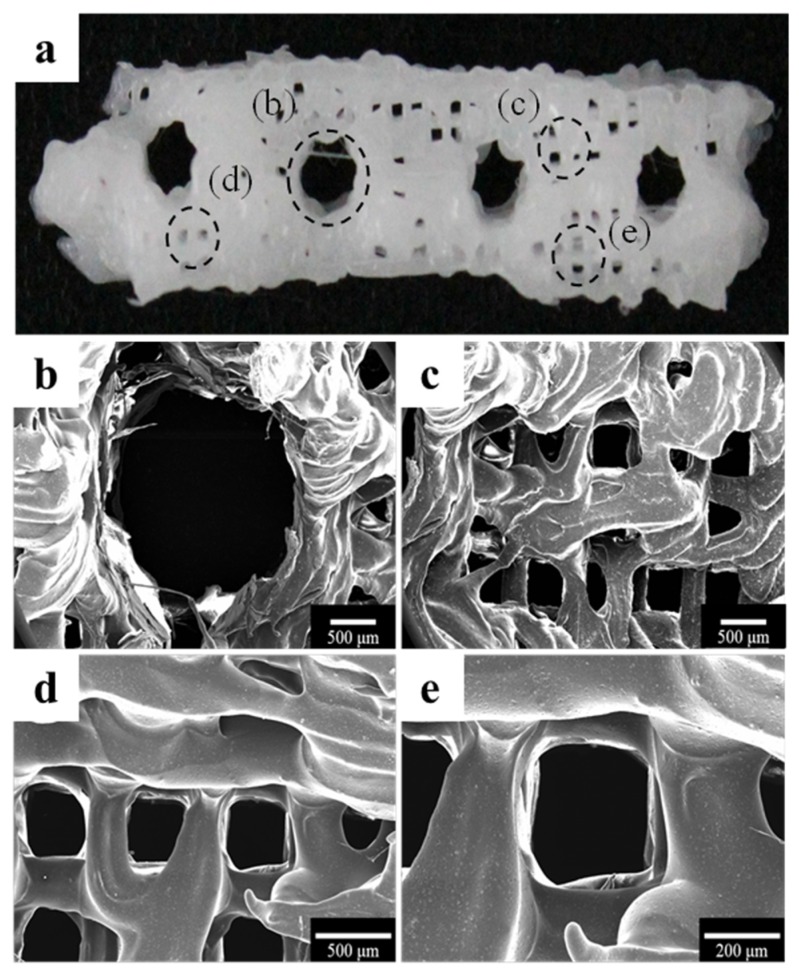
Morphology of the polycaprolactone (PCL)/β-tricalcium phosphate (β-TCP) scaffold. (**a**) Visual image of the scaffold; (**b**) An implant through hole, which plays a role in guiding the implant fixture and (**c**–**e**) The interconnected pores on the scaffold.

The morphology of the fabricated implant guide scaffold was confirmed by high-resolution field emission scanning electron microscopy (HR FE-SEM), as shown in Figure 1 and Figure 2. Figure 1a and Figure 2a show an image of the scaffold with the naked eye. Figure 1b and Figure 2b show a through hole in the scaffold that acts to guide the implant fixture. Figure 1c–e present the freeze-dried bdECM on the scaffold. The locations of the observed bdECMs shown in Figure 1c–e are indicated in Figure 1a by the dark dashed lines, respectively. The interconnected pores are observed in Figure 2c–e. The locations of the observed pores are indicated in Figure 2a by the dark dashed lines.

#### 2.1.2. Compressive Strength Comparison of PCL/*β*-TCP and PCL/*β*-TCP/bdECM Scaffolds

**Figure 3:**
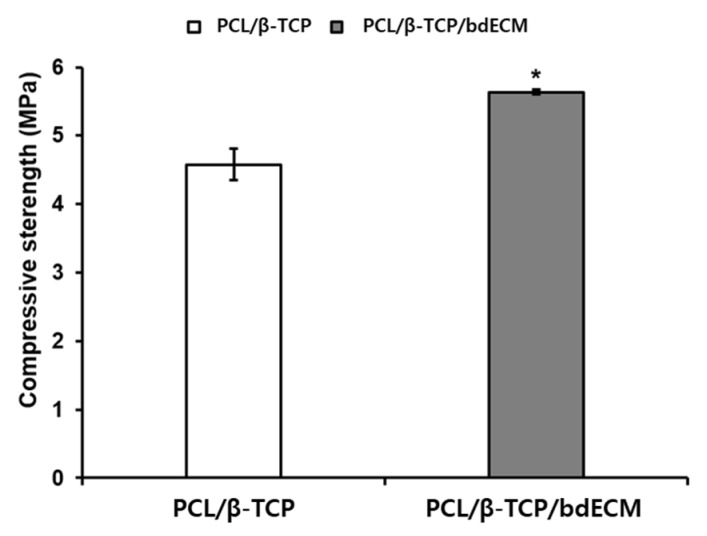
Compressive strengths of the PCL/*β*-TCP and PCL/*β*-TCP/bdECM scaffolds. The compressive strengths of the PCL/*β*-TCP and PCL/*β*-TCP/bdECM scaffolds are expressed in white and gray, respectively. In this figure, PCL/*β*-TCP/bdECM had a higher compressive strength than PCL/*β*-TCP (* *p* < 0.001).

The compressive strengths of the PCL/*β*-TCP and PCL/*β*-TCP/bdECM scaffolds were compared, as shown in Figure 3. The compressive strengths of the PCL/*β*-TCP and PCL/*β*-TCP/bdECM scaffolds were 4.575 MPa and 5.634 MPa, respectively. The PCL/*β*-TCP/bdECM scaffold had a higher compressive strength than the PCL/*β*-TCP scaffold.

#### 2.1.3. In Vitro Release Kinetics of Loaded rhBMP-2

**Figure 4:**
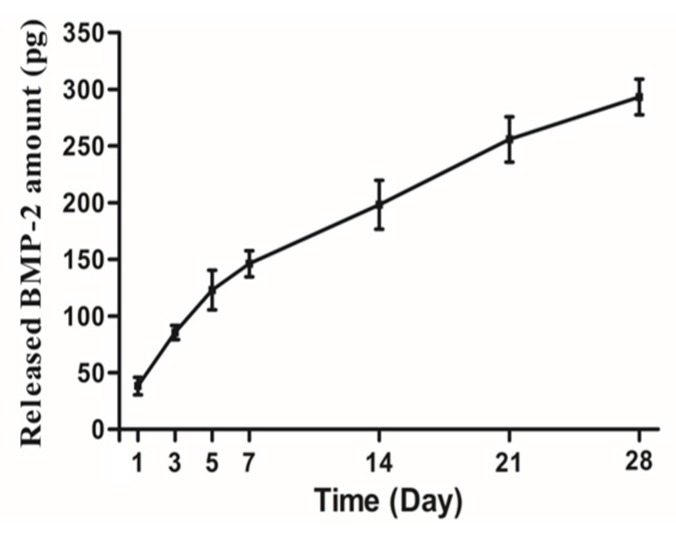
In vitro release kinetics of recombinant human bone morphogenetic protein-2 (rhBMP-2).

Figure 4 shows the amount of rhBMP-2 released from bdECM as the cumulative release. The amount of rhBMP-2 released is expressed as the cumulative release of the initial amount (1 μg). The loaded rhBMP-2 was released continuously over a 28-day period.

#### 2.1.4. In Vitro Analysis of the Cell Bioactivity

**Figure 5:**
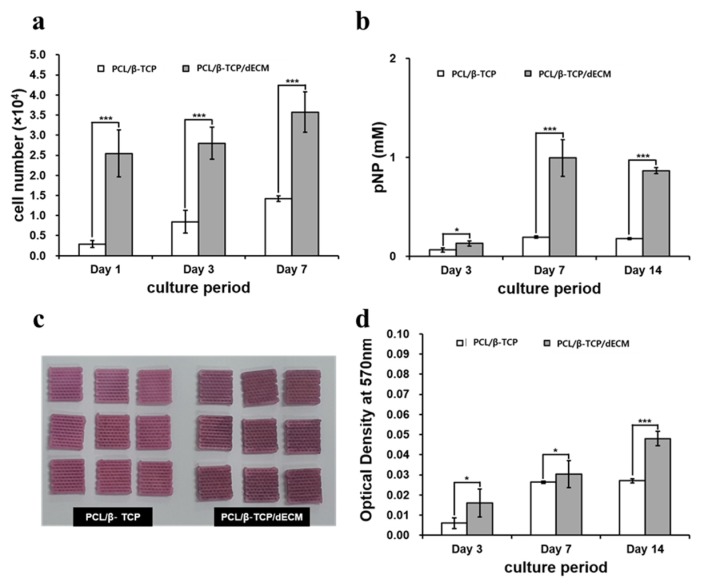
Proliferation and osteogenic differentiation assays: (**a**) The cell counting kit-8 (CCK-8) assay was used to analyze the proliferation of MC3T3-E1 cells on the scaffold; (**b**) ALP activity quantification and (**c**,**d**) alizarin red S staining was performed to evaluate osteogenic differentiation. (*: *p* < 0.05, **: *p* < 0.005, ***: *p* < 0.001)

The proliferation of MC3T3-E1 cells (a mouse pre-osteoblast cell line) on the scaffolds was investigated using the cell counting kit-8 (CCK-8) assay. The PCL/*β*-TCP/bdECM groups showed a higher initial cell adhesive ability than the PCL/*β*-TCP group at day 1 due to the additional cell binding sites from the bone bioink. These significant differences were observed up to 7 days (Figure 5a). These results suggest that the printed bone bioink enhanced the cell adhesion rate on the scaffold.

The expression of alkaline phosphatase (ALP) activity, which is regarded as an early marker of osteogenesis, and the amount of calcium deposition were measured to evaluate the extent of osteogenic differentiation. The PCL/*β*-TCP/bdECM groups showed higher ALP expression than the PCL/*β*-TCP group at day 3. PCL/*β*-TCP/bdECM showed an increase in ALP expression at day 7 and a decrease in ALP expression after day 14 (Figure 5b). These results suggest that printed bdECM containing BMP-2 promotes the initiation of osteogenic differentiation.

In alizarin red S staining, the PCL/*β*-TCP/bdECM groups that contained bone bioink and BMP-2 showed larger calcium mineral amounts compared to the PCL/TCP group during the overall observation period. In addition, the extent of calcium deposition on the scaffold was highest in the PCL/*β*-TCP/bdECM group (Figure 5c,d).

The in vitro results reveal the outstanding performance of PCL/*β*-TCP/bdECM, which exhibited cell compatibility and osteogenesis.

### 2.2. In Vivo Results

#### 2.2.1. Clinical Findings

The two experimental animals survived the surgical procedure and all implants were harvested without an inflammatory reaction. Exposure of the defect sites did not occur during the healing period and no other abnormal findings, such as infection or inflammation, were observed.

#### 2.2.2. Findings Using Micro-Computed Tomography (Micro-CT)

**Figure 6:**
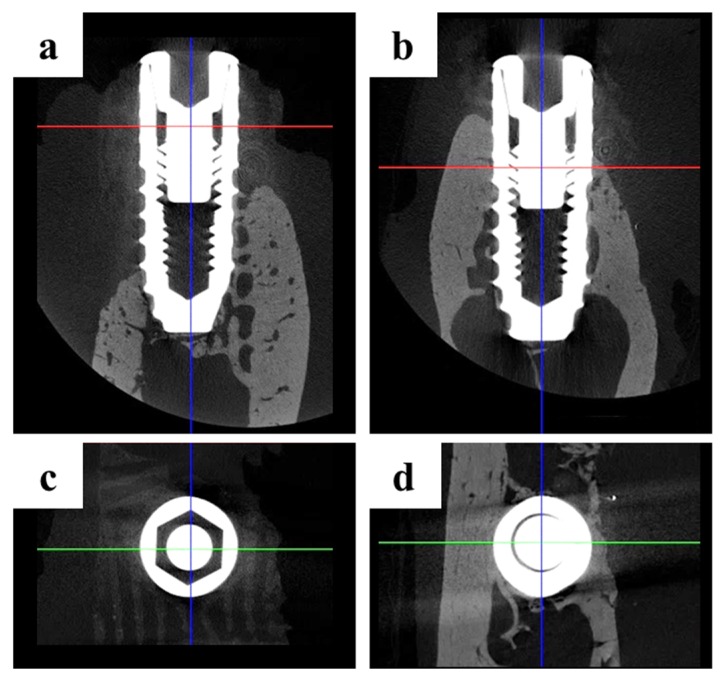
Micro-computed tomography (micro-CT) images: (**a**,**c**) Control group; (**b**,**d**) BMP group. (**a**,**b**) 3D reconstructed images of mesiodistal sections; (**c**,**d**) 3D reconstructed images of occlusal sections.

**Figure 7:**
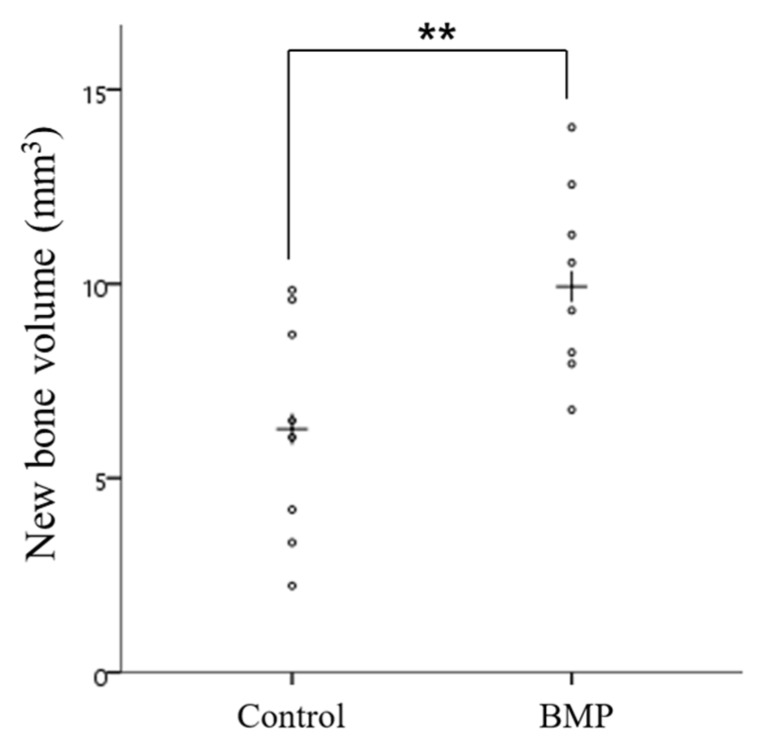
Scatter plot and median (the cross) representing new bone volume (mm^3^) (** *p* < 0.01).

Micro-CT images were obtained (Figure 6) from the scaffold that had been well positioned on the defects. The volumetric measurements were obtained using micro-CT, as summarized in Table 1 and Figure 6 and Figure 7.

The mean (± SD) new bone volume (mm^3^) in the control group and BMP group was 6.30 (±2.90) and 10.08 (±2.48), respectively. The new bone volume (mm^3^) was significantly higher in the BMP group than in the control group (*p* < 0.01).

#### 2.2.3. Histologic Findings

**Figure 8:**
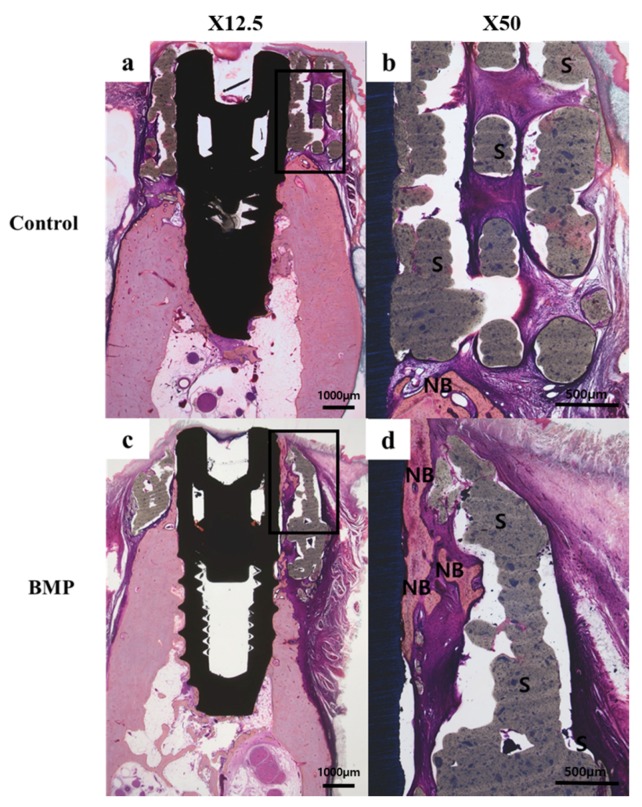
Hematoxylin-eosin stain of the histology sections: (**a**,**b**) Control group; (**c**,**d**) BMP group. **NB**: new bone, **S**: scaffold (Original magnification: 12.5× (**a**,**c**), 50× (**b**,**d**)).

**Figure 9:**
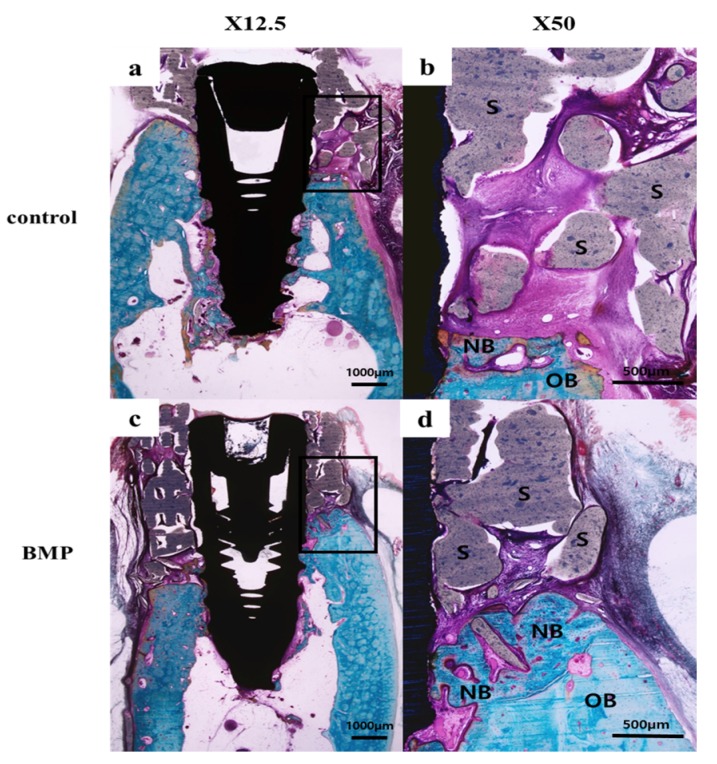
Goldner’s Masson trichrome stain of the histology sections: (**a**,**b**) Control group; (**c**,**d**) BMP group. **NB**: new bone, **OB**: old bone, **S**; scaffold (Original magnification: 12.5× (**a**,**c**), 50× (**b**,**d**)).

In all histology specimens, the scaffold was well positioned in the defect site. The tissue around the implant of all experimental groups had no other observed findings, such as inflammation. In the control group (Figure 8b and Figure 9b), fibrous and connective tissues were observed without inflammation in the entire defect site. In the BMP group (Figure 8d and Figure 9d), new bone and fibrous connective tissues were observed between the implant and the scaffold area.

#### 2.2.4. Histometric Findings

**Figure 10:**
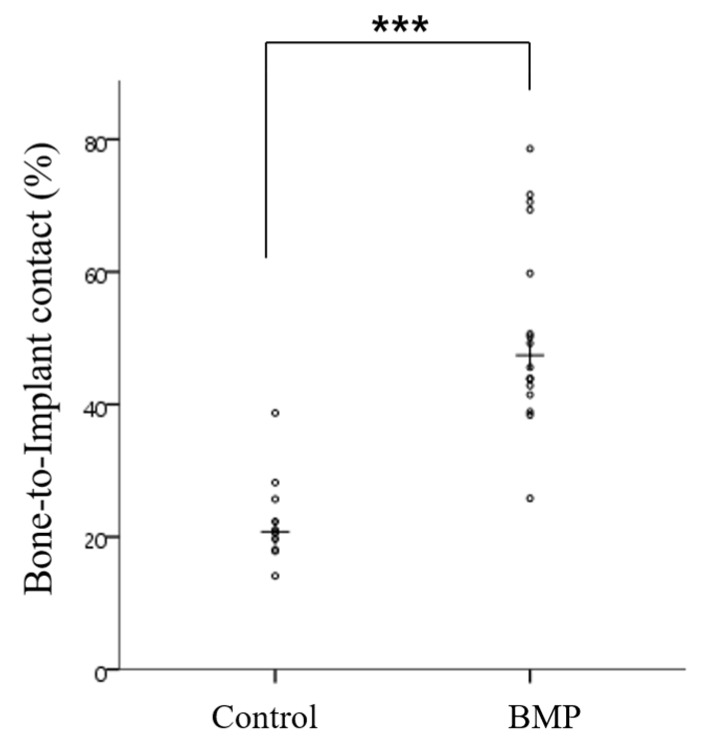
Scatter plot and median (the cross) representing the bone-to-implant contact (%) (*** *p* < 0.001).

**Figure 11:**
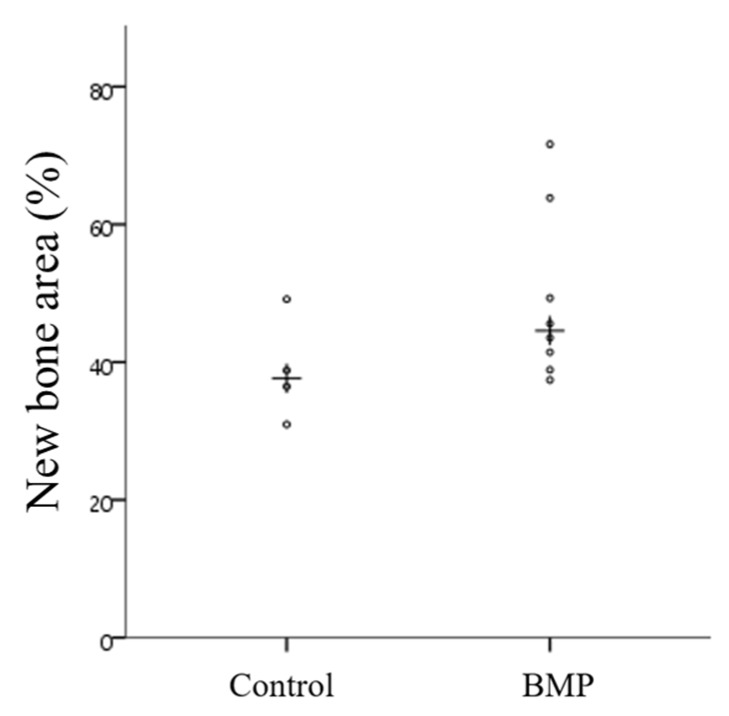
Scatter plot and median (the cross) representing the new bone area (%) (*p* > 0.05).

Table 2 and Figure 10 and Figure 11 present the histometric measurements. The control and BMP groups showed similar new bone areas (NBV; %) (*p* > 0.05). The mean (± SD) for bone-to-implant contact (%) in the control group and BMP group was 22.61 (±6.92) and 51.29 (±14.64) respectively. The bone-to-implant contact (BIC; %) was significantly higher in the BMP group than the control group (*** *p* < 0.001).

## 3. Discussion

Bone graft materials have improved considerably. On the other hand, their successful application to a vertical bone defect is limited. Limited vascular invasion and angiogenesis can lead to poor blood supply, causing necrosis or failure of the bone graft [13,14]. With the introduction of 3D-printed grafting material, it has been reported that selecting the appropriate printing materials and fabricating the desired 3D structure provide a favorable environment for angiogenesis and new bone formation [31]. The present study evaluated the osteoinductive property of 3D-printed PCL/*β*-TCP/bdECM scaffolds. The critical size defects in the mandible of an adult beagle have been reported to be 50 mm. Even if the defect in this study was smaller than the critical size, complete repair/reconstruction of the alveolar ridges rarely occurred in the large bone defects [32,33]. Therefore, this study was designed to use additional growth factors, rather than the scaffold alone, to promote new bone formation.

rhBMP-2 is an effective osteoinductive growth factor that is occasionally used in dentistry [34,35]. rhBMP-2 stimulates osteogenesis in bone defect sites during the early stages of healing through the differentiation of osteoblast, chemoattraction, angiogenesis, and cell signaling [36]. Maintaining the sustained release of rhBMP-2 from the scaffold at a constant rate is important [37]. In this study, in vitro release testing revealed bdECM to be a suitable carrier that contributes significantly to the early stage of cell binding, and maintains the consistent release of rhBMP-2.

Bioinks such as bdECM, which provide a microenvironment that is suitable to increase the cellular activities within a tissue after scaffold transplantation, have recently been studied in tissue engineering [29]. dECM is a bioink material that is used widely in transplantation procedures. The material structurally resembles natural ECM, which is composed of proteins such as glycoproteins and glycosaminoglycans (GAGs) [38,39,40]. dECM has been used in different forms, such as biological sheets and injectable hydrogels, for many years to help repair and regenerate tissue. The bioink enhances the angiogenesis to supply nutrients and oxygen in the early stages of tissue transplantation, and it also accelerates osteogenesis [28].

In the present study, rhBMP-2 was coated on a PCL/*β*-TCP/bdECM scaffold. The in vitro results showed that rhBMP-2 itself enhances osteogenic differentiation. Moreover, the cell compatibility and osteogenesis were promoted as the concentration of rhBMP-2 was increased. In vivo results have also indicated that PCL/*β*-TCP/bdECM scaffolds with rhBMP-2 conjugation enhance osteogenesis without inflammation and showed a significantly greater bone-to-implant contact ratio at the initial stage compared to the scaffold without rhBMP-2. Both the in vivo and in vitro results suggest that the transplantation of a PCL/*β*-TCP/bdECM scaffold with rhBMP-2 conjugation is biocompatible and increases the bone regeneration rate. Although osteointegration and new bone formation were observed in the rhBMP-2-conjugated PCL/*β*-TCP/bdECM scaffolds, bone regeneration in the defect exhibited a relatively low volume of new bone owing to the large dimensions of the bone defect. For the successful engraftment of 3D-printed scaffolds and subsequent bone regeneration, sufficient vascularization is needed to supply nutrients and oxygen. Insufficient vascularization in large bone defects and scaffolds may, therefore, lead to hypoxia and reduced angiogenesis [41]. Park et al. [42] attempted to resolve this problem and observed a significant improvement in vascularization and osteogenesis by the cell printing of human dental pulp stem cells (DPSCs) and vascular endothelial growth factors (VEGF). The successful results of Park et al. have provided a new perspective in the field with important implications. Nevertheless, additional studies on the use of different types of growth factors and critical defect sizes are necessary.

Another problem encountered with the low volume of new bone formation is the concentration of rhBMP-2. In this study, 400 μg/mL of rhBMP-2 was used in the conjugation, which is considered to be relatively low for activating the entire scaffold. However, several studies have suggested orthotopic and ectopic bone formation with the conjugation of rhBMP-2 and have also reported activation of a low-level systemic immune response as a side effect [43,44]. Therefore, the relatively low concentration of 400 μg/mL was used to minimize side effects while evaluating the effectiveness of rhBMP-2. Some studies have examined the appropriate concentrations of rhBMP-2. A milligram dosage (1.05–1.5 mg/mL) of rhBMP-2 was loaded onto type I collagen to achieve the osteogenesis that is comparable to the bone graft in the cleft reconstruction [45,46]. Talley et al. [47] designed a saddle defect model with the defect site measuring 7–10 mm mesiodistally, 6–8 mm apicocoronally, and 8–10 mm buccolingually; 400 μg/mL of rhBMP-2 was required to obtain sufficient new bone formation in the alveolar ridge of adult dogs [46]. A recent study using an absorbable collagen sponge (ACS) carrier coated with poly-d,l-lactic-co-glycolic acid showed that 400 μg/mL of rhBMP-2 was needed to maintain the anatomic contour of the alveolar ridge and to enhance bone formation [48]. Considering the size of the defect and the concentration of rhBMP-2 recommended in the previous studies, it was assumed that the relatively low concentration used in the present study would imply significant effectiveness. Additional studies will be needed to investigate the use of an adequate concentration of rhBMP-2 or a combination with another growth factor because the recommended concentration is already 200,000 times higher than the human physiological volume [49].

In this study, rather than using a conventional scaffold shape, the scaffold was customized and fabricated as a surgical guide to fit into the bone defect site. CBCT was used to obtain images of the bone defect site, which were used to design the scaffold that matched the shape of the bone defect and alveolar ridge and restore the original volume and shape of the alveolar bone. The scaffold had a hierarchical 400 μm-sized lattice pore network and the pores were fully interconnected to transport nutrients and promote the migration and growth of cells, ultimately improving the tissue growth efficiency [50]. Guide holes were drilled in the customized scaffold at 3 mm intervals; these were used as a surgical guide to place the fixture. The fixture itself functioned as a mechanical anchor. Therefore, no additional membrane fixing screws were required to secure the scaffold. Another advantage of this technique is that the implant placement and bone graft procedures can be done together, and no additional surgery is needed, unlike the current conventional surgical procedure.

The bone regeneration efficiency depends on factors such as the scaffold fabrication technique, materials, and types and characteristics of the biomolecules used [33,51]. The results of the micro-CT and histological analysis showed that the PCL/*β*-TCP/bdECM scaffold is an excellent candidate to promote bone regeneration in the defect site. The scaffold functioned as a good surgical guide template that led to proper implant positioning. The greatest advantage of this technique is that a single surgical procedure is needed for implantation and scaffold fixation. The placement of an implant simultaneously provides fixation between the basal bone of the bone defect site and the scaffold, and this implant and scaffold complex function as a space maintainer. The porous lattice structure of the scaffold and bdECM provides a microenvironment for bone regeneration, which enhances angiogenesis and nutrients. The coated porous scaffold releases rhBMP-2 into the pores to promote new bone formation and the early stage of osteointegration.

This study has some limitations. First, the bdECM bioink was applied to the scaffold using a centrifugation coating technique. This technique requires an additional coating process. Hence, a more simplified sequence is needed for clinical application. Three major bioprinting techniques can be used to apply 3D printing for bioinks such as bdECM: inkjet printing, laser-assisted printing, and micro-extrusion printing [52]. The micro-extrusion-based 3D printer was used to fabricate the PCL/*β* -TCP scaffold in the present study; this printer has six extrusion heads in total. Four of the six heads are connected to a heating system to melt the biomaterial, such as PCL/*β*-TCP, and the remaining two heads can be used to dispense the gel-state bone bioink. The bioink must retain its three-dimensional structure with adequate viscosity to deposit the bioink using the micro-extrusion printing technique. A high viscosity bioink may interrupt the high-resolution printing. Therefore, it is important to improve the viscosity and structural properties of bdECM bioink for the simultaneous printing of the bdECM and scaffold [52].

Several studies have investigated the technique of conjugating rhBMP-2 to the scaffold. For conjugation, the type I collagen sponge has been pre-wetted with rhBMP-2 or a freeze-drying method has been used on the printed scaffold [45,46,53]. The properties of an optimal carrier of rhBMP-2 include good bioactivity and stability and being able to control the release rate. In this study, rhBMP-2 was injected into the scaffold that had been fixed to the defect site by implant placement for intraoral applications. For accurately evaluating the effectiveness of rhBMP-2, the intraoral injection was considered since in the extraoral coating technique, performed under the water-spray cooling system, the rhBMP-2 can be washed out by saliva or by implant drilling. An advanced printing technique to uniformly print rhBMP-2 conjugated to the scaffold may be considered. The rhBMP-2 is conjugated to the bioink through a collagen microfiber and binding domain and printed with the scaffold during 3D printing, which may provide the controlled release rate of rhBMP-2 in vivo. Few studies have reported that the attachment of collagen microfibers to the bioink promotes osteogenesis [54].

## 4. Materials and Methods

### 4.1. Fabrication of the PCL/β-TCP Scaffold

#### 4.1.1. Preparation of Blended PCL/*β*-TCP

PCL (Evonik Industry, Essen, Germany) and *β*-TCP (mean diameter 100 nm, Berkeley Advanced Biomaterials Inc., Berkeley, CA, USA) were blended together by a melting process. A chip-type PCL (7 g) was placed on a glass container and melted at 120 °C for 15 min. The powdered *β*-TCP (3 g) was added to the thermally molten state of PCL. The molten-state PCL and *β*-TCP were blended for 10 min.

#### 4.1.2. Preparation of the Blended Bone-Derived Extracellular Matrix

Porcine bone was used to prepare bone bioink. Soft tissue and marrow of the porcine bone were removed manually by a surgical blade. The bone without soft tissue and marrow was freeze-dried at −85 °C for 24 h. The porcine bone was grinded using a grinder to achieve small particles of porcine bone (SB). The SB was washed with 70% ethanol to remove fat. The SB was then placed in 0.5 N hydrochloric acid (HCl) to demineralize the SB. The HCl solution was replaced every 2 h for three times to remove the undesirable particles. Demineralized SB was washed three times with distilled–deionized water (DW). Trypsin and EDTA was used to decellularize the SB. The demineralized SB was placed in a solution containing 0.05% trypsin and 0.02% EDTA for 2 h at 37 °C. To remove the trypsin and EDTA, the SB was washed three times with DW and freeze-dried at −85 °C for 24 h. A freezer-mill (6875D, SPEXSamplePrep, Metuchen, NJ, USA) was used to obtain the powdered porcine bone. Demineralized and decellularized porcine bone was pulverized using a freezer-mill. The powdered porcine bone (PB) was obtained. The bone bioink was prepared by dissolving the PB in an acidic solution containing pepsin, which enabled the use of powder-type PB as gel-state bone bioink (bdECM) [55].

#### 4.1.3. Development of a Micro Extrusion-Based 3D Printer

A micro extrusion-based 3D printer (in-house system) was developed to fabricate a tissue-engineered scaffold using solid-state synthetic biomaterial and gel-state bdECM. Six dispensing heads were installed in the system, the z-axis motion of each was individually controllable. Of the six heads, four were connected to a heating system (TCD-200EX, 901891, Iwashita Engineering, Inc., Fukuoka, Japan) to melt thermoplastic biomaterials such as polycaprolactone (PCL). The molten biomaterials were extruded by pneumatic pressure regulated by a dispenser (AD3300C, H00489, Iwashita Engineering, Inc., Fukuoka, Japan). The extruded biomaterials were cooled rapidly and hardened at room temperature. The applied temperature and pressure were adjustable up to 150 °C and 650 kPa, respectively. A linear motor, linear encoder, and linear guide were used to control the motion of the x- and y-axis, which could be operated at a maximum velocity of 2000 mm/min [56].

#### 4.1.4. Construction of a 3D Implant Model Using CAD Software

**Figure 12:**
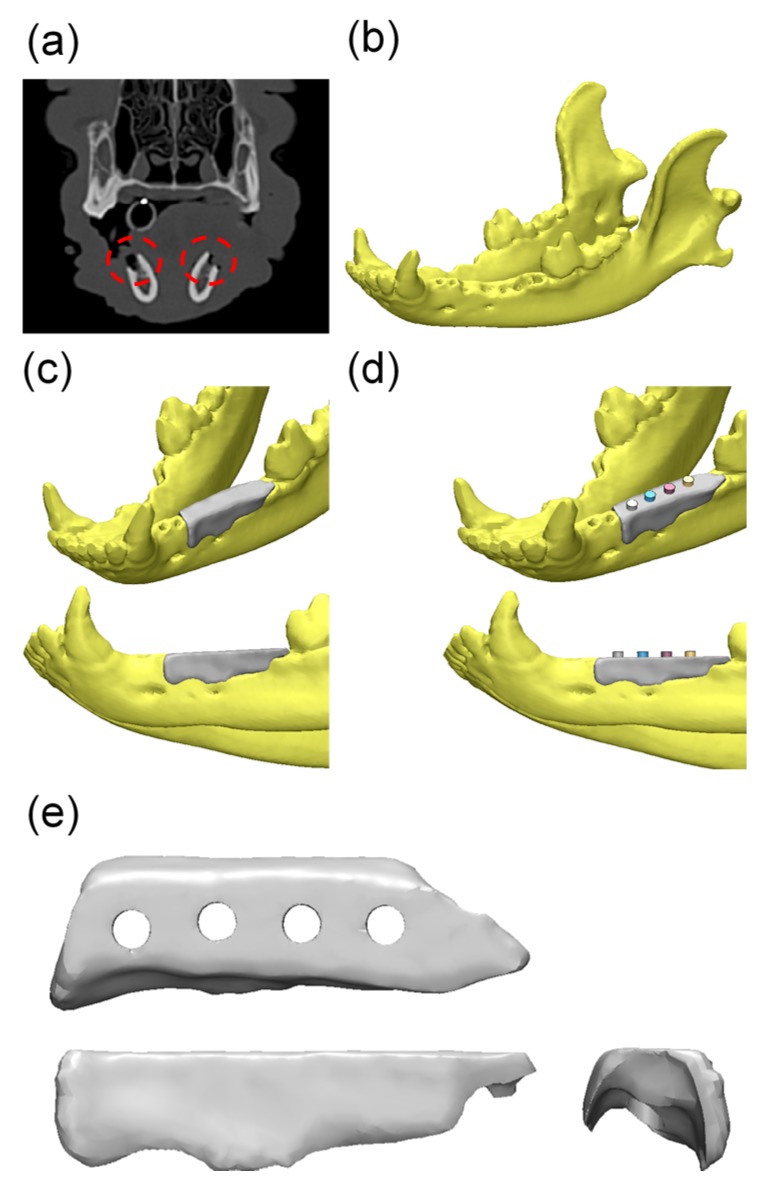
Overall modeling process of the implant guide scaffold: **(a**) Red dashed line: alveolar bone defect of mandible; **(b**) 3D modeling process of CT image; (**c**) 3D scaffold cover of the defect area; (**d**) 4 thru holes for inserting implant fixture; (**e**) Final model of implant guided scaffold.

A 3D model to fabricate the implant guide scaffold was constructed. The construction was performed using two different bio-CAD software: Mimics research (9.0 ver, Materialise, Leuven, Belgium) and 3-matic (9.0 version, Materialise, Leuven, Belgium). The Mimics research was used to apply the CT image to 3D modeling. Using sliced CT images of a beagle with a generated defect on both sides of the mandible bone, the Mimics research software reconstructed the full mandible model, as shown in Figure 12a,b. In Figure 12a, the defects generated on both sides of the mandible bone are indicated with a red dashed line. After reconstruction of the full mandible model, the model was modified and edited using the 3-matic research to design the implant guided 3D model. The model was designed to completely cover the entire area of the defect and had a customized geometry to the defect for stability of implantation. Four through holes were designed to guide the implant screws. This process is shown in Figure 11d and Figure 12c. The holes had a diameter of 2.5 mm and were arranged at 3 mm interval. The diameter of the holes was determined based on ISO 261 for implant fixtures (3.0 mm in diameter and 8.5 mm in length; TSIII, Osstem Co., Seoul, Korea). Figure 12e shows the final model of the implant guide scaffold.

#### 4.1.5. Fabrication of the PCL/*β*-TCP Scaffold for In Vitro and In Vivo Use Using an In-House System

The blended PCL/*β*-TCP was placed in a 10-mL steel syringe attached to the micro-extrusion-based 3D printer (in-house system) and the temperature was maintained at 120 °C. The 3D printing system was operated according to the code generated by the CAM software. Using the in-house system, the implant-guided PCL/*β*-TCP scaffolds for the in vivo experiments and a rectangular-type scaffold for in vitro research were fabricated. The in vivo scaffold had a geometry customized to the generated defect. On the in vivo scaffold, four through holes (diameter 2.5 mm) arranged at equal intervals (3 mm) were fabricated to guide the implant screws . The scaffolds for the in vitro tests were of two different types; the scaffolds for the compressive strength test and cell analysis were 7 mm × 7 mm × 15 mm and 7 mm × 7 mm × 1 mm, respectively. The line width, pore size, and line height of the in vitro and in vivo scaffolds were 300, 400, and 100 μm, respectively. The scaffolds had lattice-type pores; the calculated porosity was approximately 57% and the pores were fully interconnected. The fabricated scaffold was washed with 70% ethanol to remove any undesirable burrs or particle.

#### 4.1.6. Preparation of bdECM with rhBMP-2

rhBMP-2 (Cowellmedi, Busan, Korea) and bdECM were blended by a mixing process. To blend the powder-type rhBMP-2 and gel-state bdECM, 200 μg of rhBMP-2 was dissolved in 500 μL distilled–deionized water and mixed well using a vortex mixer (G560E, Scientific Industry, Inc., Bohemia, NY, USA). The rhBMP-2 solution was added to 4.5 mL of the bdECM and mixed manually for 2 min.

#### 4.1.7. The Coating Process of the in Vitro and in Vivo Scaffolds Using bdECM

The scaffolds for the in vivo and in vitro experiments were coated with bdECM to use the material as a carrier. Gel-state bdECM and the scaffold were placed in a 10 mL plastic syringe attached to the centrifuge. The centrifuge was operated according to rpm, time, and temperature. The pre-set conditions were maintained at 1200 rpm, 5 min, and 4 °C, respectively. After the centrifugal process, the scaffolds and bdECM were placed in a 37 °C incubator to reticulate the bone bioink. The coated scaffolds were freeze-dried at −85 °C for 24 h. In the case of the scaffold for the in vitro experiment with 7 mm × 7 mm × 1 mm dimensions, the volume of the coated bdECM and amount of rhBMP-2 loaded per scaffold were calculated. Because this scaffold was used as a sample in the release kinetics test and cell analysis, the calculated volume of the coated bdECM and loaded rhBMP-2 per scaffold were approximately 29 μL and 1 μg, respectively. The coated scaffolds were sterilized with a 450 W UV lamp for 2 h.

#### 4.1.8. Scanning Electron Microscope Analysis of the Scaffolds 

The morphology of the implant guide scaffolds was observed by high-resolution field emission scanning electron microscopy (HR FE-SEM, Nova NanoSEM200, FEI Co., Hillsboro, OR, USA) at 10 kV. The scaffolds were coated with platinum for 3 min using a sputter coater.

### 4.2. In Vitro Test with Scaffold

#### 4.2.1. Compressive Strength Test Using PCL/*β*-TCP and PCL/*β*-TCP having bdECM

The compressive strength test was measured to compare PCL/*β*-TCP and PCL/*β*-TCP/bdECM scaffolds and determine if the coated bdECM enhances the compressive strength. A universal testing machine (3304, Instron, Norwood, MA, USA) was used in the test. The compressive module for the test was equipped in the testing machine. The test was conducted under several conditions. The temperature of the testing room was 25 °C and the humidity was 30%. The moving velocity of the testing machine was 1 mm/min [57,58,59].

#### 4.2.2. In Vitro Release Kinetics of rhBMP-2

The release kinetics of the rhBMP-2 loaded in bdECM was confirmed using an ELISA kit (DBP200; R&D Systems, Minneapolis, MN, USA). In this experiment, the 7 mm × 7 mm × 1 mm PCL/*β*-TCP/bdECM scaffold was used. The scaffold was placed in a 6-well plate with phosphate buffer saline (PBS). The 6-well plate was incubated at 37 °C. A soup of PBS (1 mL) was collected at 1, 3, 5, 7, 14, 21, and 28 days. After collection, fresh PBS (1 mL) was added to maintain constant volume.

#### 4.2.3. Cell Culture and Seeding of MC3T3-E1

The cell culture of mouse pre-osteoblasts (MC3T3-E1) was conducted using α-MEM (12571071; Gibco, Rockville, MD, USA) containing 10% FBS and 1% penicillin/streptomycin (15140122; Gibco, Rockville, MD, USA). The cells were incubated under physiological conditions (37 °C, 5% CO_2_). The culture medium was replaced every 2 days. To conduct the proliferation assay, the cells were seeded at a density of 1 × 10^5^ cells per scaffold and cultured in the same medium used for the culture process. For the osteogenic differentiation assay, the cells were seeded at a density of 3 × 10^5^ cells per scaffold and cultured in an osteogenic medium (α-MEM containing 20% FBS, 10^−8^ M dexamethasone, 0.2 mM ascorbic acid, 10 mM β-glycerol phosphate (Sigma Aldrich, Saint Louis, MO, USA) and 1% penicillin/streptomycin).

#### 4.2.4. Analysis of Proliferation and Osteogenic Differentiation of MC3T3-E1

A cell counting kit-8 (CCK-8, Dojindo, Rockville, MD, USA) was used to analyze the proliferation of MC3T3-E1 on the scaffold at each time point. The time points for proliferation were 1, 3, and 7 days after seeding. Using the culture medium, the CCK-8 solution was diluted 1:10 and added to each sample. The samples were incubated for 3 h at 37 °C. After incubation, the culture supernatant was extracted and the optical density at 450 nm was measured using a microplate reader (Epoch, BioTek, Winooski, VT, USA). P-nitrophenyl phosphate (pNPP; Sigma-Aldrich, Saint Louis, MO, USA) was used to quantify the ALP activity of the MC3T3 cells on days 3, 7, and 14 after seeding. The samples were lysed in RIPA lysis buffer and incubated in a pNPP solution at 37 °C for 30 min. After incubation, the microplate reader was used at 405 nm for quantification using p-nitrophenol standard. With using alizarin red staining, calcium deposition on the scaffold was estimated at 3, 7, and 14 days. Samples were fixed with 4% paraformaldehyde and stained with 2% alizarin red solution (pH 4.2) for 10 min at room temperature. A 10% cetylpyridinium chloride solution was used to extract the alizarin red stain from the samples. The extraction was measured at 570 nm using a microplate reader.

### 4.3. In Vivo Study

#### Experimental Animals and Design

Two groups were prepared in this study: one experimental group, and one control group. In two dogs, the mandibular premolars, P1, P2, P3 and P4 were extracted bilaterally. After two weeks of healing, four standardized bone defects were prepared on each side of the mandible and four implants (3.0 mm in diameter and 8.5 mm in length; TSIII, Osstem Co., Seoul, Korea) were placed at each defect. The scaffolds were produced and used after the formation of the defects. In the experimental group, rhBMP-2 (400 μg/mL, CowellMedi, Busan, Korea) was injected into the scaffold using syringes. In the control group, no other materials were added.

The study was approved by the Chonnam National University Animal Experimental Ethics Committee (CNU IACUC-YB-2016-43). Two beagle dogs, which were fed and raised according to the protocol of Chonnam National University, were chosen for the study. Two dogs, each two years old and approximately 13–15 kg in weight, were chosen for the study after two weeks of adaptation and feeding a soft dog food diet. Healthy beagle dogs without periodontal disease were selected.

The study group is as follows:

1. Control group (Defect: 2 (fixture: 8): PCL/*β*-TCP/bdECM scaffold.
2. BMP group (Defect: 2 (fixture: 8): PCL/*β*-TCP/bdECM scaffold + rhBMP-2 (400 μg/mL, total volume/defect 200 uL).

### 4.4. Design of Surgical Surgery

#### 4.4.1. Tooth Extraction

Atropine sulfate (0.05 mg kg^−1^ IM; Dai Han Pharm Co., Seoul, Korea) was administered orally once before surgery, and general anesthesia was performed with isoflurane (Choongwae, Seoul, Korea). The electrocardiogram was monitored continuously during surgery. Additional infiltration anesthesia was performed with lidocaine (1 mL; Yu-Han Co., Gunpo, Korea) containing 1:100,000 epinephrine in the buccal and lingual mucosa of the surgical site.

In the first operation, the teeth were extracted from the mandible of the beagle and a defect was formed on the day of extraction. Computed tomography (Siemens Emotion 16; Siemens, Munich, Germany) was performed at 120 mAs and 130 kV at 1 mm slice thickness. The incision site was sutured with 4-0 Vicryl (Mersilk, Ethicon Co., Livingston, UK) to allow healing. Immediately after surgery, a broad spectrum antibiotic (penicillin G procaine and penicillin G benzathine) was administered and an intramuscular injection (1 mL/5 kg) was administered 48 h later. Amoxicillin (20 mg/kg) and previrus (5 mg/kg) were administered orally for two weeks after the operation, and enrofloxacin (10 mg/Kg) was injected subcutaneously for three days. N/S flushing and oral gel were applied daily until the end of the study.

#### 4.4.2. Produce Scaffold for Defect Size

**Figure 13:**
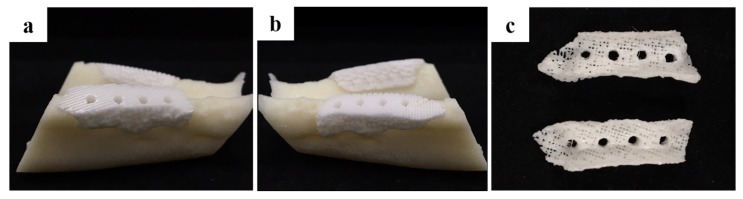
Photograph of a PCL/*β*-TCP/bdECM scaffold used in the in vivo animal studies: (**a**) Right side view; (**b**) Left side view; (**c**) Inferior view.

After the formation of the defect, CT scans were taken and a scaffold was made within one week. The scaffold was made to match the shape of the defect and extraction socket. For scaffold construction, a hole for the fixture was formed and a 2.5 mm diameter hole in the scaffold was made considering the fixture size (Figure 13). The average length of the scaffold was 26.35 mm. After confirming that it fitted the plaster model, it was sterilized and used. Considering the vertical length of the mandible, a scaffold was made with a total vertical length of 4 to 5 mm, leaving a minimum vertical bone length of 3 mm.

#### 4.4.3. Sacrifice after Implant Placement

**Figure 14:**
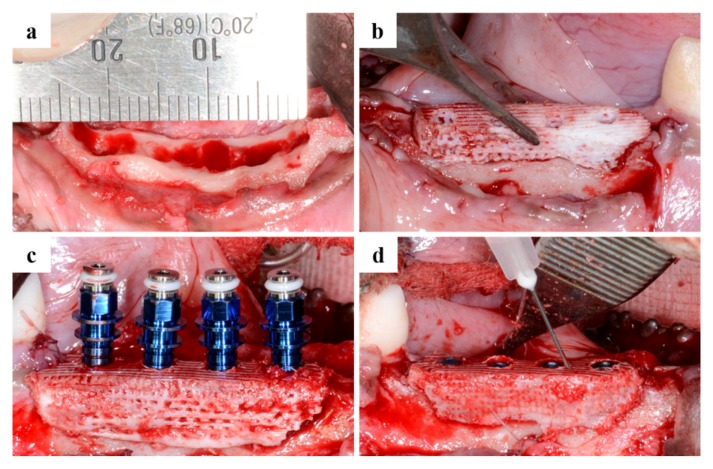
Surgical procedures: (**a**) The defect area after tooth extraction; (**b**) A scaffold was placed in the defect sites; (**c**) Progress of implant placement; (**d**) Application of the prepared BMP containing a medium.

After two weeks, atropine sulfate (0.05 mg kg^−1^ IM; Dai Han Pharm Co., Seoul, Korea) was administered and anesthesia was maintained with isoflurane (Choongwae, Seoul, Korea) gas anesthesia. Lidocaine, containing 1:100,000 epinephrine (1 mL; Yu-Han Co., Gunpo, Korea), was injected into the mucosa of the surgical site for surgery. In this operation, a pre-fabricated scaffold was placed at the defect site, followed with implant placement. A total of 16 implants (3.0 mm in diameter and 8.5 mm in length; TSIII, Osstem, Seoul, Korea) were used. In the BMP group, rhBMP-2 (400 μg/mL; CowellMedi, Busan, Korea) was injected carefully around the scaffold after implant placement (Figure 14). In the control group, only the scaffold was applied to the defect site, which was sutured after implant placement.

The postoperative mucosal health, exposure to the surgical site, tissue necrosis, and infection were monitored daily. The suture was removed one week after implant placement. The dogs were sacrificed three months after the operation.

#### 4.4.4. Micro-Computed Tomography (µCT) Analysis

**Figure 15:**
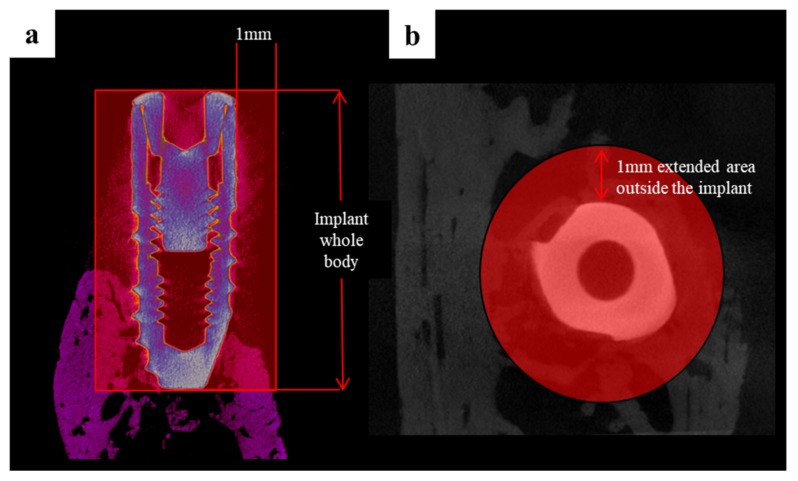
The region of interest in micro-computed tomography (micro-CT) images: (**a**) Coronal section view; (**b**) Horizontal section view.

Harvested mandibles were wrapped with film (Parafilm M, Bemis Company, Inc., Neenah, WI, USA) to prevent evaporation during the scans, which were performed using the following settings. Scanning was performed at a 130 kV energy, 60 μA intensity, and a 7.10 μm pixel resolution using a Bruker micro-CT (SkyScan-1173 version 1.6, Bruker; Kontich, Belgium). All scan parameters were identical for all the specimens. The region of interest (ROI) is the width of the expanded area outside the implant (1 mm), and the height is the whole implant body (Figure 15).

#### 4.4.5. Histological Analysis

All specimens were fixed in neutral buffered formalin (Sigma Aldrich, St. Louis, MO, USA) for two weeks and dehydrated sequentially in ethanol (70%, 80%, 90% and 100%). The dehydrated specimens were embedded in Technovit 7200 resin (Heraeus KULZER, South Bend, IN, USA) to form blocks. The specimen block was cut longitudinally from the implant center using an EXAKT diamond cutter (KULZER EXAKT 300, EXAKT, Norderstedt, Germany).

**Figure 16:**
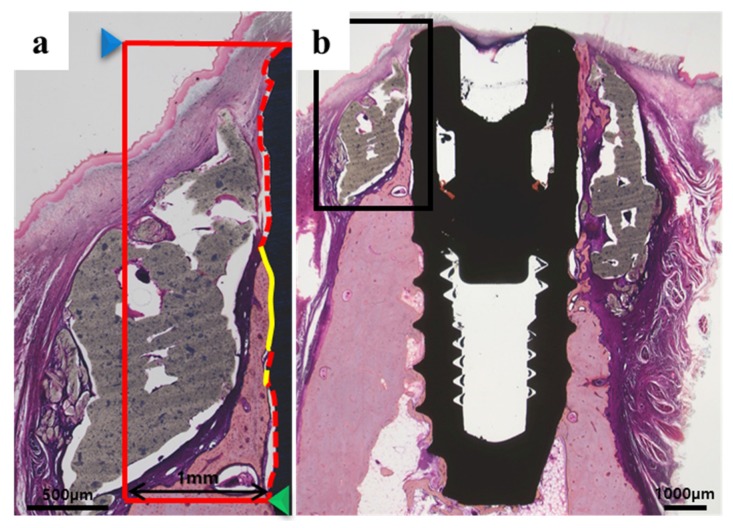
Bone-to-implant contact and new bone area in histologic specimens: (**a**) Red box, the area of interest (AOI is 1 mm wide and height from the new bone to the top of the implant (NB-I)); Blue arrow, top of implant; Green arrow, the bottom of new bone (NB); Yellow line, bone-to-implant contact (BIC).The following measurements were analyzed within the histologic specimens; (**b**) Representative histologic specimens.

The slides were prepared by grinding a section using an EXAKT grinding machine (KULZER EXAKT 400CS, EXAKT, Norderstedt, Germany) to a thickness of 30 μm. Histological staining was performed with hematoxylin-eosin and Goldner’s Masson trichrome. Images were taken using a computer and an optical microscope (Olympus BX, Tokyo, Japan) attached to a CCD camera (Polaroid DMC2 digital microscope camera (Polaroid Corporation, Cambridge, MA, USA)). The bone-to-implant contact area (BIC; %) and new bone area (NBA; %) were measured using an image analysis program (i-solution, IMT, Vancouver, BC, Canada). A 50× magnification was used for histometric analysis and the overall specimen image was captured at a 12.5× magnification (Figure 16).

1. New bone area (NBA, %) = New bone Area/Area of interest × 100.
2. Bone-to-implant contact (BIC, %) = length of bone-to-implant contact/length from the top of the implant to the bottom of new bone (NB) × 100.

### 4.5. Statistical Analysis

All experimental data are expressed as means, standard deviations, and medians. Statistical software R (version 3.2.5, R Foundation, Vienna, Austria) was used for all statistical analysis. Brunner and Langer (2000) [60] was used to compare the histomorphometrical and micro-CT results between the groups. The statistical significance level was set to 5%.

## 5. Conclusions

This preliminary study was conducted to evaluate the simultaneous use of an implant surgical guide and a bone graft of the 3D-printed PCL/*β*-TCP/bdECM scaffold conjugated with rhBMP-2, which restored the original volume and shape of the alveolar ridge in the defect site. This scaffold performed well as a surgical guide to place the implant at the proper location and depth. The in vitro results revealed higher cell adhesive ability and sustained rhBMP-2 release, and the in vivo results indicated that the growth factor of the scaffold had integrated the new bone formation around the implant well. Within the limitations of this preliminary study, fixation of the PCL/*β*-TCP/bdECM scaffold conjugated with rhBMP-2 can be an easy and effective technique for simultaneous implant placement and bone grafting in a large bone defect site. On the other hand, further large-scale studies will be needed to confirm these results.

## Figures and Tables

**Table 1:** New bone volume within the area of interest (*n* = 16; mm^3^).

Group	Mean ± SD	Median	*p*-Value
Control	6.30 ± 2.90	6.26	<0.01
BMP	10.08 ± 2.48	9.93	

**Table 2:** Histometric analysis within the area of interest (*n* = 16; %).

Variable	Group	Mean ± SD	Median	*p*-Value
BIC (%)	Control	22.61 ± 6.92	20.78	<0.001 ***
	BMP	51.29 ± 14.64	47.41	
NBA (%)	Control	38.84 ± 7.61	37.63	0.073
	BMP	48.95 ± 12.35	44.57	

NBA: new bone area; BIC: bone-to-implant contact.
